# Monocytes Infiltrate the Pancreas via the MCP-1/CCR2 Pathway and Differentiate into Stellate Cells

**DOI:** 10.1371/journal.pone.0084889

**Published:** 2014-01-08

**Authors:** Kazuko Ino, Masahiro Masuya, Isao Tawara, Eri Miyata, Keiko Oda, Yoshiki Nakamori, Kei Suzuki, Kohshi Ohishi, Naoyuki Katayama

**Affiliations:** Department of Hematology and Oncology, Mie University Graduate School of Medicine, Tsu, Mie, Japan; University of Leuven, Rega Institute, Belgium

## Abstract

Recent studies have shown that monocytes possess pluripotent plasticity. We previously reported that monocytes could differentiate into hepatic stellate cells. Although stellate cells are also present in the pancreas, their origin remains unclear. An accumulation of enhanced green fluorescent protein (EGFP)^+^CD45^–^ cells was observed in the pancreases and livers of chimeric mice, which were transplanted with a single hematopoietic stem cell isolated from EGFP-transgenic mice and treated with carbon tetrachloride (CCl_4_). Because the vast majority of EGFP^+^CD45^–^ cells in the pancreas expressed stellate cell-associated antigens such as vimentin, desmin, glial fibrillary acidic protein, procollagen-I, and α-smooth muscle actin, they were characterized as pancreatic stellate cells (PaSCs). EGFP^+^ PaSCs were also observed in CCl_4_-treated mice adoptively transferred with monocytes but not with other cell lineages isolated from EGFP-transgenic mice. The expression of monocyte chemoattractant protein-1 (MCP-1) and angiotensin II (Ang II) increased in the pancreas of CCl_4_-treated mice and their respective receptors, C-C chemokine receptor 2 (CCR2) and Ang II type 1 receptor (AT1R), were expressed on Ly6C^high^ monocytes isolated from EGFP-transgenic mice. We examined the effect of an AT1R antagonist, irbesartan, which is also a CCR2 antagonist, on the migration of monocytes into the pancreas. Monocytes migrated toward MCP-1 but not Ang II *in vitro*. Irbesartan inhibited not only their *in vitro* chemotaxis but also *in vivo* migration of adoptively transferred monocytes from peripheral blood into the pancreas. Irbesartan treatment significantly reduced the numbers of EGFP^+^F4/80^+^CCR2^+^ monocytic cells and EGFP^+^ PaSCs in the pancreas of CCl_4_-treated chimeric mice receiving EGFP^+^ bone marrow cells. A specific CCR2 antagonist RS504393 inhibited the occurrence of EGFP^+^ PaSCs in injured mice. We propose that CCR2^+^ monocytes migrate into the pancreas possibly via the MCP-1/CCR2 pathway and give rise to PaSCs.

## Introduction

Monocytes are bone marrow (BM)-derived circulating leukocytes and precursors for tissue macrophages and dendritic cells [1]. Recent studies demonstrated that monocytes differentiated into non-hematopoietic cells such as endothelial progenitor cells and keratinocyte-like cells [2], [3]. We previously reported that monocytes could become hepatic stellate cells (HpSCs) during carbon tetrachloride (CCl_4_)-induced injury [4]. In the course of a study using chimeric mice transplanted with a single hematopoietic stem cell isolated from enhanced green fluorescent protein (EGFP)-transgenic mice [5], we detected EGFP^+^ hematopoietic stem cell-derived cells in the pancreas. Therefore, we examined the cell fate of these transplanted EGFP^+^ cells in the pancreas of chimeric mice, and found that hematopoietic stem cell-derived cells may partially contribute to the generation of pancreatic stellate cells (PaSCs). EGFP^+^ PaSCs were also detected in CCl_4_-treated mice adoptively transferred with monocytes isolated from EGFP-transgenic mice.

Monocyte chemoattractant protein-1 (MCP-1) is a family member of C-C chemokines and is produced by various cell types including fibroblasts, endothelial cells, smooth muscle cells, keratinocytes, hepatocytes, monocytes/macrophages, and lymphocytes in response to proinflammatory molecules such as tumor necrosis factor-α, interferon-γ, and lipopolysaccharide [6]–[9]. C-C chemokine receptor 2 (CCR2), a high-affinity receptor for MCP-1, is expressed on many hematopoietic cell types such as hematopoietic progenitor cells, lymphocytes, and monocytes/macrophages [10]–[12]. The MCP-1/CCR2 pathway is involved in the development of inflammation and fibrosis in many organs including liver, pancreas, skin, heart, and kidney [13]–[17].

Local renin-angiotensin-system (RAS) exists in peripheral tissues such as kidney, heart, liver, and pancreas [18]. The main bioactive component of RAS is angiotensin II (Ang II), which is derived from angiotensinogen by renin and Ang-converting enzyme [19]. Ang II participates in the regulation of cell growth and inflammatory responses [20]. Two subtypes of Ang II receptors, type 1 (AT1R) and type 2 (AT2R), have been identified and both receptors are detected in a wide variety of cell types including hematopoietic cells [19]–[23]. The majority of Ang II-induced physiological and pathological effects are mediated by AT1R.

Both MCP-1 and Ang II are known to promote the migration of hematopoietic cells toward sites of inflammation [11], [24]–[27]. We investigated the roles of the MCP-1/CCR2 pathway and Ang II/AT1R pathway in the recruitment of hematopoietic stem cell-derived cells from the circulation into the pancreas using an AT1R antagonist, irbesartan, which also acts as an antagonist of CCR2 because of its molecular structure [28]. We observed that monocytes migrated toward MCP-1 but not Ang II *in vitro*, and that irbesartan inhibited their migration. The numbers of EGFP^+^F4/80^+^CCR2^+^ monocytic cells and EGFP^+^ PaSCs significantly decreased in the pancreas of CCl_4_-injured chimeric mice receiving EGFP^+^ BM total nucleated cells (TNCs) after treatment with irbesartan. A highly selective CCR2 antagonist RS504393 also inhibited *in vitro* Ly6C^+^ monocyte migration toward MCP-1 and *in vivo* occurrence of EGFP^+^ PaSCs in the pancreas of the injured mice. These data suggest that CCR2^+^ monocytes are likely to migrate into the pancreas via the MCP-1/CCR2 pathway and give rise to PaSCs *in situ*.

## Materials and Methods

### Mice

Breeding pairs of C57BL/6J-Ly5.1 mice were purchased from Jackson Laboratories (Bar Harbor, ME, USA). Breeding pairs of EGFP-transgenic mice (C57BL/6-Ly5.2 background) were kindly provided by Dr. M. Okabe (Osaka University, Japan) [29]. Mice were bred and maintained at the Institute of Laboratory Animals, Mie University. All experimental procedures were approved by the Institutional Animal Care and Use Committee of Mie University (approval number 22–40) and were performed according to the Mie University guidelines for laboratory animals.

### Antibodies and Cytokines

Described in Materials and Methods S1.

### Cell Preparation

Ten- to 12-week-old male EGFP-transgenic mice were used as BM and peripheral blood (PB) donors. BM-TNCs were flushed from femurs and tibiae, and then washed twice with Ca^2+^/Mg^2+^-free phosphate-buffered saline (PBS^–^, Gibco, Grand Island, NY, USA) containing 0.1% deionized fraction V bovine serum albumin (Sigma-Aldrich, St. Louis, MO, USA). BM-mononuclear cells (MNCs) were isolated by gradient separation using Lympholyte-M (Cedarlane, Hornby, Canada), and lineage marker negative (Lin^–^) cells were prepared by negative selection using anti-CD4, anti-CD8a, anti-CD45R/B220, anti-Gr-1, anti-Mac-1 and anti-TER-119 antibodies, and immunomagnetic beads (Dynabeads M-450 coupled to sheep anti-rat immunoglobulin G (IgG), Dynal, Great Neck, NY, USA). The resulting Lin^–^ cells were stained with phycoerythrin (PE)-anti-Sca-1, allophycocyanin (APC)-anti-c-kit, biotinylated anti-CD34 and mouse lineage panel antibodies, followed by streptavidin-APC-cyanine (Cy) 7. CD34^–^c-kit^+^Sca-1^+^Lin^–^ (CD34^–^KSL) cells were enriched by fluorescence-activated cell sorting with a FACSAria sorter (BD Biosciences, San Diego, CA, USA). For clonal cell transplantation, using a single-cell deposition device on a FACSAria, single CD34^–^KSL cells were deposited into round-bottomed 96-well plates (Corning, Corning, NY, USA) containing α-minimum essential medium, 20% fetal bovine serum (Hyclone Laboratories, Logan, UT, USA), 1% deionized fraction V bovine serum albumin, 1×10^−4^ mol/l 2-mercaptoethanol, 100 ng/ml steel factor and 100 ng/ml interleukin-11. The plates were incubated at 37°C in a humidified atmosphere with 5% CO_2_. At 18 hours after single-cell deposition, the wells containing single cells were marked and then incubated for 7 days. We selected clones consisting of no more than 20 cells after incubation for clonal cell transplantation.

Monocytic lineage cells were isolated from BM cells using a two-step method as described previously [4]. Monocyte-enriched cells were prepared from BM-MNCs by negative selection using anti-CD4, anti-CD8a, anti-CD45R/B220, anti-Ly6G, anti-TER119, anti-c-kit, anti-Sca-1, anti-Thy-1.2 and anti-CCR3 antibodies, and sheep anti-rat IgG-coated Dynabeads M450. For adoptive transfer experiments, the resulting cells were stained with PE-anti-CD45.2, APC-anti-c-kit and biotinylated anti-Ly6C antibodies, followed by streptavidin-APC-Cy7. CD45.2^+^Ly6C^high^c-kit^–^ cells were enriched by fluorescence-activated cell sorting with a FACSAria sorter. For flow cytometric analysis, RT-PCR analysis, and chemotaxis assay, the resulting cells were stained with PE-anti-Ly6C antibody and anti-PE microbeads, and Ly6C^+^ monocytes were enriched by immunomagnetic cell selection.

Whole blood was drawn by heart puncture, and PB-TNCs were isolated by lysis of red cells with ACK lysis buffer. In one experiment, Ly6C^+^ cells were depleted from PB-TNCs by immunomagnetic cell selection using PE-anti-Ly6C antibody and anti-PE microbeads.

### Transplantation

Ten- to 12-week-old male C57BL/6J-Ly5.1 mice were irradiated with a single 950 cGy dose of total body irradiation using a 4×10^6^ V linear accelerator. Clonal cell transplantation was performed using a modified method as previously described [5], [30]. The contents of wells containing 20 or fewer viable clusters of cells derived from single CD34^–^KSL cells were injected into the tail veins of irradiated C57BL/6J-Ly5.1 mice together with 2×10^5^ Sca-1^–^ BM-MNCs obtained from C57BL/6J-Ly5.1 mice, which served as radioprotective cells during the post-irradiation pancytopenia period.

To prepare BM-TNC-transplanted mice, we intravenously injected 2×10^6^ BM-TNCs obtained from male EGFP-transgenic mice into irradiated male C57BL/6J-Ly5.1 mice. All chimeric recipient mice exhibited high levels of multilineage engraftment (92–95%).

### Flow Cytometric Analysis

For analysis of hematopoietic engraftment, PB was obtained from the retro-orbital plexus of recipient mice using heparin-coated micropipettes (Drummond Scientific, Broomall, PA, USA). Red blood cells were lysed with ACK lysis buffer. The percentage of donor-derived cells (EGFP^+^ cells) among B cell, T cell and myeloid lineages was analyzed by staining with PE-anti-CD45R/B220, PE-anti-Thy-1.2, and a combination of PE-anti-Gr-1 and PE-anti-Mac-1 antibodies, respectively.

To analyze the expression of CCR2 or AT1R on Ly6C^+^ monocytes, cells were stained with anti-CCR2 or anti-AT1R antibodies, followed by Alexa Fluor 647-conjugated secondary antibodies. Analysis was performed using a FACSCalibur (BD Biosciences).

### Experimental Design

**Figure 1:**
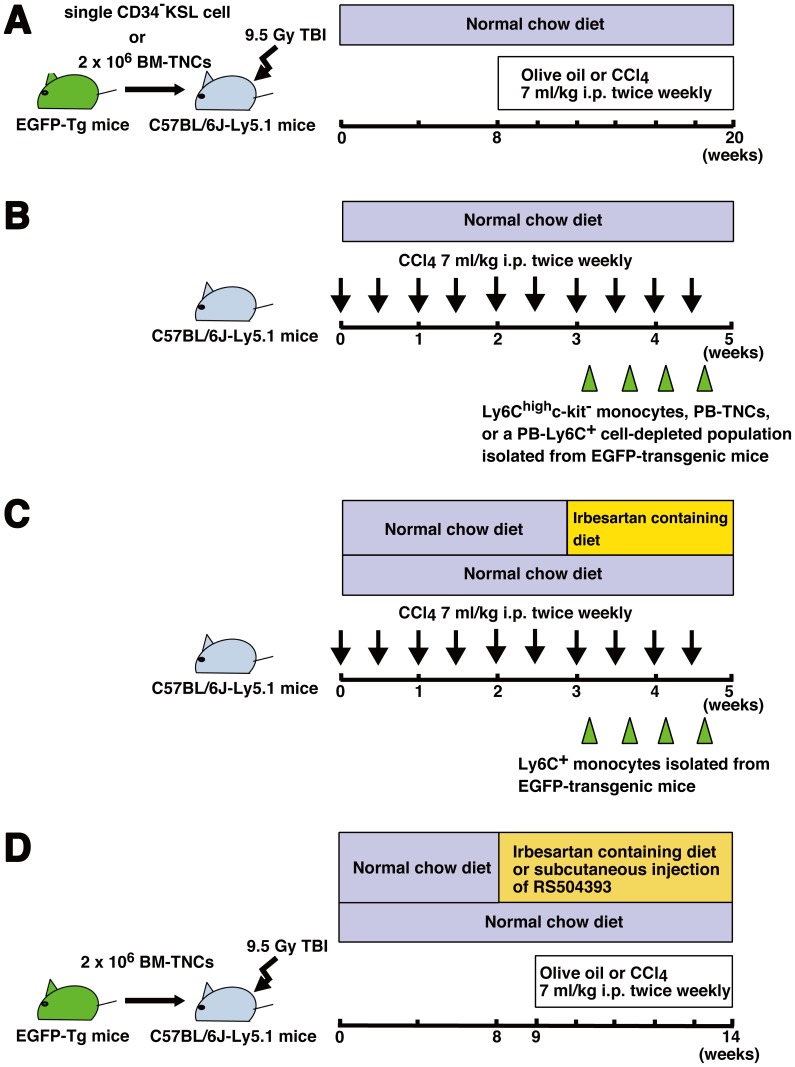
Experimental design. (A) Clones from a CD34^–^c-kit^+^Sca-1^+^lineage^–^ (CD34^–^KSL) cell or a total of 2×10^6^ bone marrow-total nucleated cells (BM-TNCs) isolated from enhanced green fluorescent protein (EGFP)-transgenic mice were transplanted into lethally irradiated C57BL/6J-Ly5.1 mice. Two months after BM transplantation, mice were intraperitoneally injected with CCl_4_ or olive oil twice a week for 12 weeks. (B) C57BL/6J-Ly5.1 mice were intraperitoneally injected with CCl_4_ twice weekly for 5 weeks. Ly6C^+^ monocytes, peripheral blood (PB)-TNCs, or a PB-Ly6C^+^ cell-depleted population isolated from EGFP-transgenic mice were adoptively transferred into CCl_4_-treated mice at 24 hours after each injection of CCl_4_ for 2 weeks. (C) C57BL/6J-Ly5.1 mice were intraperitoneally injected with CCl_4_ twice weekly for 5 weeks. They were fed chow containing irbesartan or normal chow for 2 weeks. Ly6C^+^ monocytes isolated from EGFP-transgenic mice were adoptively transferred into CCl_4_-treated mice at 24 hours after each injection of CCl_4_ for 2 weeks. (D) C57BL/6J-Ly5.1 mice that received 2×10^6^ EGFP^+^ BM-TNCs were fed chow containing irbesartan or normal chow for 6 weeks. In another group of mice, RS504393 or vehicle was subcutaneously administered once a day for 6 weeks. One week after the initiation of irbesartan or RS504393 treatment, CCl_4_ treatment was started and continued for 5 weeks.

In the first experiment, starting at 8 weeks after BM transplantation, mice that received single EGFP^+^CD34^–^KSL cell-derived cells or 2×10^6^ EGFP^+^ BM-TNCs were intraperitoneally injected with 7 ml/kg body weight of olive oil alone or the same amount of a 1∶4 solution of CCl_4_ (Wako Pure Chemical Industries, Osaka, Japan) and olive oil twice a week for 12 weeks. Mice were euthanized at 48 hours after the last injection of CCl_4_ (Figure 1A).

In the second experiment, 8-week-old male C57BL/6J-Ly5.1 mice were intraperitoneally injected with CCl_4_ twice weekly for 5 weeks. Starting at the seventh dose of CCl_4_, we intravenously administered Ly6C^high^c-kit^–^ monocytes, PB-TNCs, or a PB-Ly6C^+^ cell-depleted population isolated from EGFP-transgenic mice into CCl_4_-treated mice at 24 hours after each injection of CCl_4_ for 2 weeks. Mice were euthanized at 48 hours after the last injection of cells (Figure 1B).

In the third experiment, 8-week-old male C57BL/6J-Ly5.1 mice were intraperitoneally injected with CCl_4_ twice weekly for 5 weeks. Starting at 3 weeks after CCl_4_ treatment, mice were fed chow containing the AT1R antagonist, irbesartan (30 mg/kg/day, kindly provided by Dainippon-Sumitomo Pharmaceutical, Osaka, Japan) or normal chow for 2 weeks. Two days after the initiation of irbesartan treatment, we intravenously administered Ly6C^+^ monocytes isolated from EGFP-transgenic mice into CCl_4_-treated mice at 24 hours after each injection of CCl_4_ for 2 weeks. Mice were euthanized at 48 hours after the last injection of cells (Figure 1C).

In the fourth experiment, we used mice that received EGFP^+^ BM-TNCs. Starting at 8 weeks after BM transplantation, recipient mice were fed chow containing irbesartan (30 mg/kg/day) or normal chow for 6 weeks. In another group of mice, we also subcutaneously administered RS504393, a selective CCR2 antagonist (2 mg/kg/day, Tocris Bioscience, Ellisville, MO) or dimethyl sulfoxide once a day for 6 weeks. One week after the initiation of irbesartan or RS504393 treatment, mice were treated with CCl_4_ for 5 weeks. Mice were euthanized at 48 hours after the last injection of CCl_4_ (Figure 1D).

### Tissue Processing

Mice were anesthetized by inhalation of isoflurane and an intraperitoneal injection of pentobarbital, and then perfused with 25 ml PBS^–^ followed by 25 ml 4% phosphate-buffered paraformaldehyde (Wako Pure Chemical Industries) via the left ventricle, before pancreases were isolated by dissection. Pancreases were fixed with 4% phosphate-buffered paraformaldehyde for 1 hour at room temperature. Some tissue blocks were embedded in paraffin after dehydration in a graded alcohol series. Other tissue blocks were fixed with 4% phosphate-buffered paraformaldehyde for another 4 hours and then embedded in Tissue-Tek OCT compound (Sakura Finetek USA, Torrance, CA, USA), rapidly frozen in liquid nitrogen, and then stored at −80°C until they were cryosectioned. Tissue blocks were cut to 5-µm sections using a microtome or cryostat.

### Immunohistochemical Analysis

Frozen sections were treated with 0.5% Triton X-100 (Wako Pure Chemical Industries) in PBS^–^ for 1 hour and then washed three times with PBS^–^ for 5 minutes. To detect hematopoietic lineage cells, frozen sections were incubated with purified rat anti–mouse CD45 followed by Alexa Fluor 568-conjugated goat anti-rat IgG or PE-conjugated antibodies against hematopoietic lineage markers. As negative controls, immunohistostaining without the primary antibody or with PE-conjugated isotype control was performed. Nuclei were stained with TO-PRO-3 iodide (Molecular Probes Invitrogen, Eugene, OR, USA). Triple-fluorescence immunohistochemistry was performed by incubating frozen sections with three combinations of antibodies: (1) anti-vimentin, anti-glial fibrillary acidic protein (GFAP), anti-desmin or anti-procollagen 1A1, followed by Alexa Fluor 568-conjugated secondary antibodies and APC-anti-CD45; (2) after treatment with a M.O.M. Immunodetection Kit (Vector Laboratories, Burlingame, CA, USA), Cy3-anti-α-smooth muscle actin (α-SMA) and APC-anti-CD45; or (3) anti-CCR2 followed by Alexa Fluor 568-donkey anti-goat IgG and APC-anti-F4/80. Staining without the primary antibody and staining with appropriate APC- or Cy3-conjugated isotype controls were performed as negative controls. All sections were examined under an Olympus IX81 FV1000 laser scanning confocal microscope.

### RT-PCR

Described in Materials and Methods S1.

### Chemotaxis Assay

Isolated monocytes were assayed for their ability to migrate through pores in a polycarbonate membrane towards various concentrations of MCP-1 or Ang II in a 24-well transwell plate (5-µm pore size, Corning). RPMI 1640 with 0.2% fetal bovine serum was used as the assay medium. Prior to experiments, irbesartan and RS504393 were dissolved in dimethyl sulfoxide (vehicle) to prepare a concentrated stock solution. A volume of 600 µl assay medium containing MCP-1 (0.1 or 1 nmol/l) or Ang II (1 or 10 µmol/l) was added to the lower chamber. The filter membrane was sandwiched between upper and lower chambers, and 100 µl of cell suspensions (2×10^6^ cells/ml) was added to the upper chamber. After incubation at 37°C in a humidified atmosphere with 5% CO_2_ for 3 hours, non-migrating cells were removed with a cotton tip, and the migrated cells on the filter membrane were fixed, stained with Diff-Quick solution (Sysmex, Kobe, Japan) and counted in 20 high-power fields (magnification×400) under an Olympus BX-41 light microscope. In some experiments, the assay was performed with monocytes preincubated with either irbesartan (1, 10, 20 or 50 µmol/l), RS504393 (0.1 or 1 µmol/l), or vehicle only, at 37°C in a humidified atmosphere with 5% CO_2_ for 1 hour. Then, the preincubated monocytes were added to the upper chamber and 1 nmol/l MCP-1 or 100 ng/ml macrophage inflammatory protein-1α (MIP-1α) was added to the lower chamber. The plates were then incubated for 3 hours. All conditions were evaluated in triplicate for each experiment.

### Statistical Analysis

Data are expressed as the means ± SD. The statistical significance was assessed by unpaired Student’s *t*-test and Mann-Whitney *U* test. A value of *P*<0.05 was considered statistically significant.

## Results

### Expression of Markers for PaSCs in EGFP^+^CD45^−^ Cells Derived from a Single Hematopoietic Stem Cell

**Figure 2:**
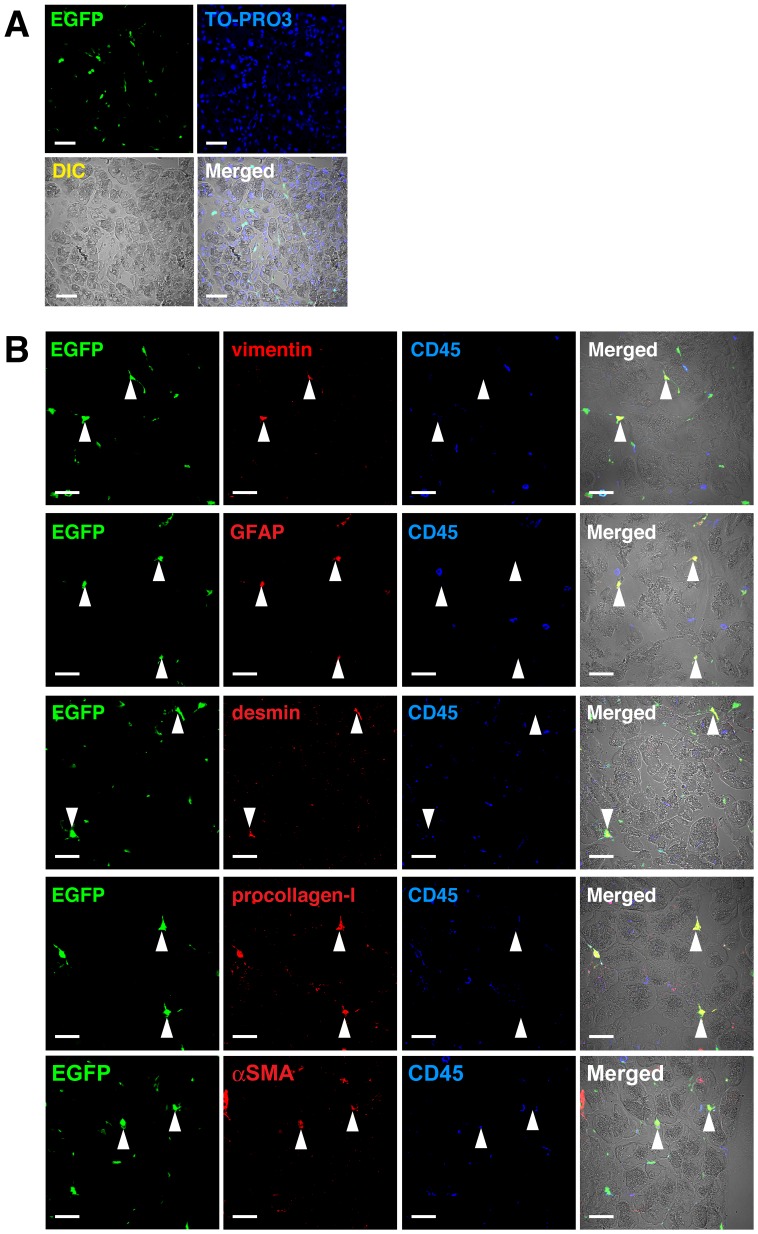
Expression of PaSC-associated antigens in EGFP^+^ cells in pancreas of mice receiving clonal cell populations derived from a single EGFP^+^ hematopoietic stem cell. (A) Pancreases from CCl_4_-treated mice that received clones from a CD34^–^KSL cell isolated from EGFP-transgenic mice were examined immunohistochemically. Panels show EGFP as green, TO-PRO-3 nuclei stain as blue, differential interference contrast (DIC) image, and the combined merged image. Scale bars, 30 µm. (B) Panels show EGFP as green, vimentin, GFAP, desmin, procollagen-I or α-SMA as red, CD45 as blue, and the merged images of these markers together with DIC images. White triangles indicate EGFP^+^CD45^–^vimentin^+^, EGFP^+^CD45^–^GFAP^+^, EGFP^+^CD45^–^desmin^+^, EGFP^+^CD45^–^procollagen-I^+^ or EGFP^+^CD45^–^α-SMA^+^ cells. Scale bars, 30 µm.

Previously, we reported that hematopoietic stem cell-derived cells could differentiate into HpSCs during CCl_4_ injury in chimeric mice (mouse 1–3 in Table 1) transplanted with clonal populations of cells derived from a single EGFP^+^CD34^–^KSL cell [5]. CCl_4_ was reported to cause histological changes in the pancreas such as subcapsular and interlobar edema [31]. We examined whether EGFP^+^ cells existed in the pancreas of two chimeric mice (Figure 1A). These mice (mouse 1 and 2 in Table 1) revealed high-level multilineage engraftment. As shown in Figure 2A, we also detected EGFP^+^ cells in the pancreas of these chimeric mice. Because PaSCs share many morphological and functional characteristics with HpSCs [32], we stained pancreas sections with anti-CD45 antibody and antibodies against stellate cell-associated antigens. As shown in Figure 2B, cells positive for stellate cell-associated antigens were all negative for CD45. Almost 30% of EGFP^+^ cells were negative for CD45 and two thirds of EGFP^+^CD45^–^ cells were positive for vimentin, GFAP, and desmin (Table 2). Approximately 50% and 40% of EGFP^+^CD45^–^ cells were positive for procollagen-I and α-SMA, respectively. The frequencies of CD45^–^ cells among EGFP^+^ cells and cells positive for stellate cell-associated antigens among EGFP^+^CD45^–^ cells in the chimeric mice (n = 2) were almost identical to those in mice (n = 3) that had received EGFP^+^ BM-TNC transplants (Table 2). These results suggested that PaSCs are partially derived from hematopoietic stem cells in the CCl_4_-treated mice.

**Table 1:** Hematopoietic engraftment in mice transplanted with clonal cells derived from a single CD34^–^KSL cell.

	Chimerism, % EGFP^+^ cells		
Mouse	Gr-1^+^/Mac-1^+^	B220^+^	Thy-1.2^+^
1	86	86	28
2	69	78	36
3	30	46	12
4	13	10	4

EGFP, enhanced green fluorescent protein; CD34^–^KSL cell,

CD34^–^c-kit^+^Sca-1^+^lienage^–^ cell.

**Table 2:** PaSC expression markers in donor-derived cells from injured pancreas of mice transplanted with clonal cells derived from a single CD34^–^KSL cell or BM-TNC.

	CD45^–^ cells inEGFP^+^ cells, %	CD45^–^ vimentin^+^cells in EGFP^+^cells, %	CD45^–^ GFAP^+^ cellsin EGFP^+^ cells, %	CD45^–^ desmin^+^cells in EGFP^+^cells, %	CD45^–^ procollagen-I^+^cells in EGFP^+^cells, %	CD45^–^ α-SMA^+^ cellsin EGFP^+^cells, %
**Mice transplanted with clonal cells derived from a single CD34^–^KSL cell (n = 2)**						
No. 1	36.2	27.8	28.3	27.1	27.3	14.9
No. 2	30.6	12.1	14.6	19.3	7.8	10.2
mean	33.4	20.0	21.5	23.2	17.6	12.6
**Mice transplanted with BM-TNCs (n = 3)**						
No. 1	29.4	19.5	16.0	25.5	15.0	10.8
No. 2	31.5	17.6	18.0	18.9	10.1	6.2
No. 3	33.8	17.3	26.0	16.6	21.8	13.7
mean ± SD	31.6±2.2	18.1±1.2	20.0±5.3	20.3±4.6	15.6±5.9	10.2±3.8

PaSC, pancreatic stellate cell; CD34^–^KSL cell, CD34^–^c-kit^+^Sca-1^+^lienage^–^ cell; BM-TNCs, bone marrow-total nucleated cells; EGFP, enhanced green fluorescent protein; GFAP, glial fibrillary acidic protein; α-SMA, α-smooth muscle actin; SD, standard deviation. We analyzed the pancreases of two mice transplanted with clonal cells derived from a single CD34^–^KSL cell and three mice transplanted with BM-TNCs.

### Adoptive Transfer of Ly6C^+^ Monocytes into CCl_4_-treated Mice

**Figure 3:**
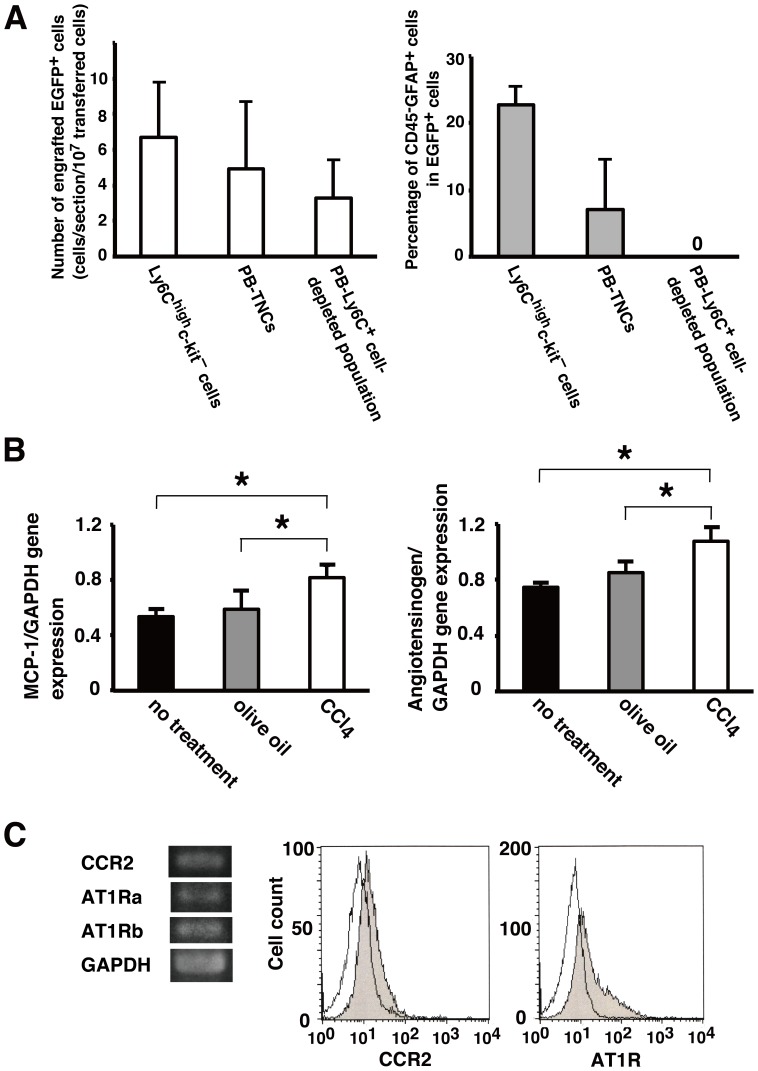
MCP-1 and angiotensinogen production in pancreas of CCl_4_-treated mice and their receptor expression on monocytes. (A) The number of engrafted EGFP^+^ cells and the percentages of CD45^–^GFAP^+^ cells among EGFP^+^ cells in the pancreases of mice that received Ly6C^high^c-kit^–^ cells, PB-TNCs, or a PB-Ly6C^+^ cell-depleted population isolated from EGFP-transgenic mice are shown. Data are the means ± SD of three mice. (B) Total RNA isolated from the pancreases from four untreated mice, four olive oil-treated mice, and three CCl_4_-treated mice was analyzed by RT-PCR for mRNA expression of MCP-1 and angiotensinogen. The level of mRNA was normalized to that of GAPDH mRNA. Data are the means ± SD of three or four mice per group. *P<0.05 versus untreated mice or olive oil-treated mice. (C) Total RNA from Ly6C^+^ monocytes, which were isolated from the BM of naive EGFP mice, was analyzed by RT-PCR for mRNA expression of CCR2, AT1Ra, and AT1Rb. Representative examples of CCR2, AT1Ra, and AT1Rb mRNA expression are shown in the left panel. The expression of CCR2 and AT1R on Ly6C^+^ monocytes was evaluated by single-color flow cytometry. Representative examples of histograms for CCR2 and AT1R expression are shown in the center and right panel, respectively. Black lines indicate isotype control staining.

Our previous study demonstrated that HpSCs were derived from monocytes using adoptive transfer of various isolated lineages of mature blood cells into CCl_4_-treated mice [4]. We intravenously transferred Ly6C^high^c-kit^–^ monocytes, which were isolated from the BM of EGFP-transgenic mice, into non-irradiated CCl_4_-treated mice (Figure 1B). Because the recovery of adoptively transferred EGFP^+^ cells in the pancreas was very low, the total number of EGFP^+^ cells in 10 sections of the pancreas was counted. In the pancreas of mice (n = 3) that received monocytes, the number of EGFP^+^ cells engrafted in the pancreas was 6.7±3.1 cells/section/10^7^ transferred cells and the percentage of CD45^–^GFAP^+^ cells among EGFP^+^ cells was 22.8±2.7% (Figure 3A). To confirm that monocytes were direct precursors of PaSCs, we adoptively transferred PB-TNCs and a PB-Ly6C^+^ cell-depleted population containing ≤0.5% monocytes into CCl_4_-treated mice. As shown in Figure 3A, the number of EGFP^+^ cells engrafted in the pancreas was 4.9±3.8 cells/section/10^7^ transferred cells and the percentage of CD45^–^GFAP^+^ cells among EGFP^+^ cells was 7.1±7.6% in the pancreas of mice (n = 3) that received EGFP^+^ PB-TNCs. Although the number of EGFP^+^ cells in the pancreas of mice (n = 3) that received the EGFP^+^ PB-Ly6C^+^ cell-depleted population was 3.3±2.2 cells/section/10^7^ transferred cells, there were no EGFP^+^CD45^–^ cells in the pancreas. These findings support the possibility that a number of engrafted Ly6C^+^ monocytes differentiated into PaSCs in the pancreas.

### Production of MCP-1 and Angiotensinogen in the Pancreas and Monocyte Expression of CCR2 and AT1R

MCP-1 and Ang II have been implicated in the development of pancreatitis and pancreatic fibrosis [33]–[35]. Moreover, it was reported that CCl_4_ injury induces the production of MCP-1 and angiotensinogen in the liver [36]. Based on these findings, we analyzed the expression of MCP-1 and angiotensinogen in the pancreases of untreated mice, olive oil-treated mice, and CCl_4_-treated mice. Higher expression of MCP-1 and angiotensinogen was observed in the pancreases of CCl_4_-treated mice, compared with untreated mice or olive oil-treated mice (Figure 3B).

Next, we isolated Ly6C^+^ monocytes from the BM of naive EGFP mice, and investigated the expression of CCR2 and AT1R on these cells by RT-PCR and flow cytometry (Figure 3C). In rodents, two AT1R isoforms have been identified: AT1Ra and AT1Rb [37]. mRNA expression of CCR2, AT1Ra, and AT1Rb was determined by RT-PCR and flow cytometric analysis revealed the expression of cell surface CCR2 and AT1R.

### Migration of Ly6C^+^ Monocytes via the MCP-1/CCR2 Pathway, but not the Ang II/AT1R Pathway

**Figure 4:**
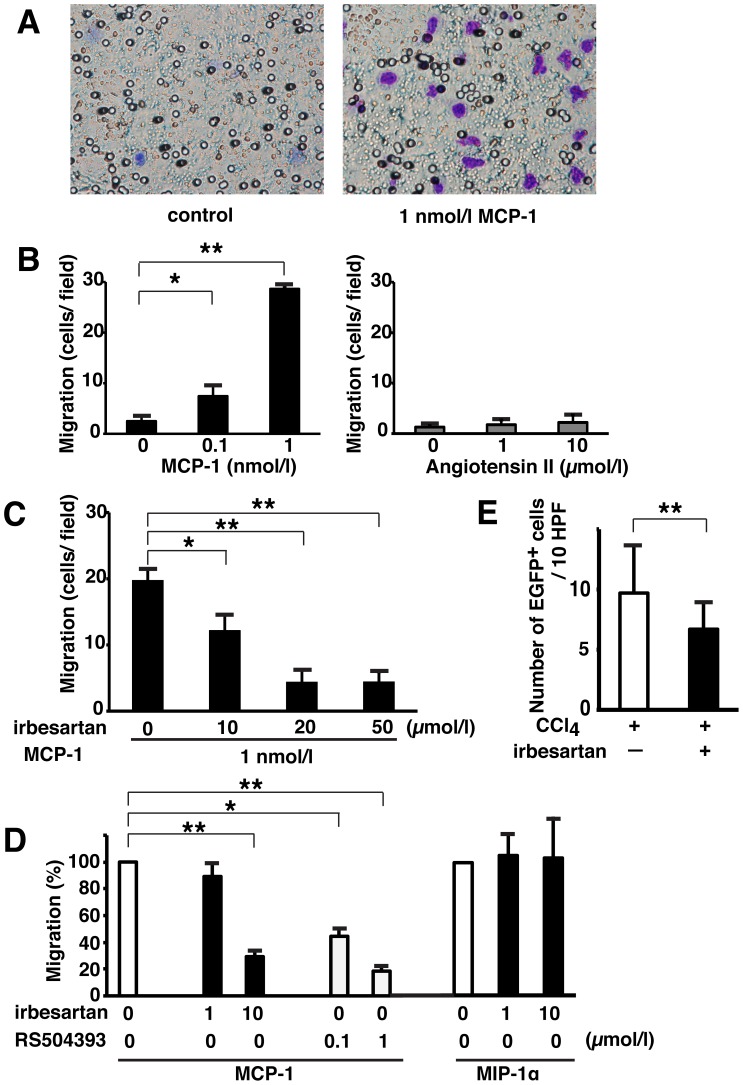
Inhibitory effect of irbesartan on Ly6C^+^ monocyte migration toward MCP-1. Chemotaxis of Ly6C^+^ monocytes toward MCP-1, Ang II, or MIP-1α was investigated using transfilter assays. (A) Photos show representative micropore membrane images with or without MCP-1 (1 nmol/l) in the lower chamber of a 24-well transwell plate. (B) Ly6C^+^ monocytes (2×10^5^) were seeded in the upper chamber of a 24-well transwell plate, and various concentrations of MCP-1 or Ang II (0.1 and 1 nmol/L or 1 and 10 µmol/L, respectively) were placed in the lower chamber. Data are the means ± SD of three wells and representative of two independent experiments. *P<0.05; **P<0.01 versus control. (C) Effect of various concentrations of irbesartan on Ly6C^+^ monocyte migration towards MCP-1 (1 nmol/l). Data are the means ± SD of three wells and representative of two independent experiments. *P<0.05; **P<0.01 versus control. (D) Comparison of irbesartan and RS504393 for Ly6C^+^ monocyte migration. Ly6C^+^ monocytes were treated with the indicated concentrations of irbesartan or RS504393 for 1 hour and subjected to MCP-1- or MIP-1α-induced chemotaxis. Data are the means ± SD of three wells and representative of two independent experiments. *P<0.05; **P<0.01 versus control. (E) Effects of irbesartan on migration of adoptive transferred EGFP^+^ monocytes into the pancreas of CCl_4_-treated mice. Number of engrafted EGFP^+^ cells in 10 sections of pancreas from mice treated with or without irbesartan is shown. Data are the means ± SD of three different mice per group. *P<0.05 versus control mice fed a normal chow.

Both MCP-1 and Ang II are associated with the migration of monocytes toward sites of inflammation [11], [24], [25]. To clarify whether the MCP-1/CCR2 pathway or Ang II/AT1R pathway, either alone or together, were associated with monocyte infiltration into the pancreas, we examined the effects of irbesartan, an AT1R antagonist with CCR2 antagonizing properties, on the *in vitro* chemotactic migration of Ly6C^+^ monocytes isolated from BM. Chemotaxis of Ly6C^+^ monocytes towards MCP-1 or Ang II was investigated using transfilter assays in 24-well transwell plates. We used naive Ly6C^+^ monocytes isolated from unstimulated EGFP-transgenic mice. Figure 4A shows representative micropore membranes, which illustrate the effects of 1 nmol/l MCP-1 on Ly6C^+^ monocyte migration. As shown in Figure 4B, MCP-1 stimulated Ly6C^+^ monocyte migration, with 11.4-fold induction at 1 nmol/l MCP-1 compared with controls. Although Ly6C^+^ monocytes expressed AT1R, Ang II did not stimulate their migration (1.7-fold induction at 10 µmol/l Ang II vs. control), in contrast to previous reports [24], [25]. Irbesartan attenuated MCP-1-induced Ly6C^+^ monocyte migration dose-dependently, and a maximal inhibition of migration was observed at 20 µmol/l irbesartan (Figure 4C). As shown in Figure 4D, a highly selective CCR2 antagonist RS504393 [38] also dramatically inhibited MCP-1-induced monocyte migration with a similar inhibitory effect to that of irbesartan. However, irbesartan did not inhibit MIP-1α-induced monocyte migration. Next, we examined the effect of irbesartan on *in vivo* migration of Ly6C^+^ monocytes isolated from the BM of naïve EGFP mice into the pancreas of CCl_4_-treated mice using an adoptive transfer model (Figure 1C). In mice administered an irbesartan (30 mg/kg body weight/day)-containing diet, the migration of EGFP^+^ monocytes into the pancreas was significantly inhibited (Figure 4E). These results indicated that the MCP-1/CCR2 pathway may play an important role in the migration of Ly6C^+^ monocytes from the circulation to the injured pancreas.

### Effects of Irbesartan on the Occurrence of Hematopoietic Cell-derived PaSCs in the Pancreas of CCl_4_-treated Mice

**Figure 5:**
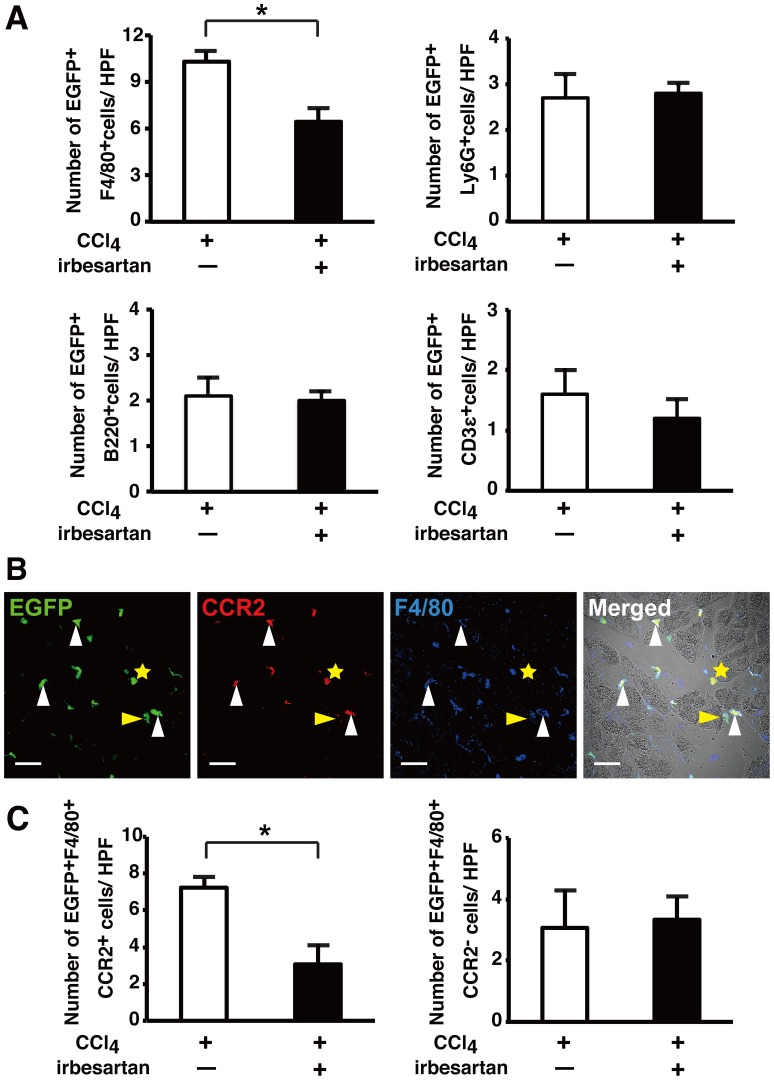
Effect of irbesartan on hematopoietic lineage cell infiltration into the CCl_4_-injured pancreas. EGFP^+^ BM-TNC-transplanted mice were fed a normal chow diet or an irbesartan-containing diet and treated with CCl_4_ for 6 weeks. (A) Numbers of EGFP^+^F4/80^+^ monocytic cells, EGFP^+^Ly6G^+^ neutrophils, EGFP^+^B220^+^ B cells, and EGFP^+^CD3ε^+^ T cells in the pancreas of both groups. Data are the means ± SD of three mice per group. *P<0.05 versus mice fed a normal chow. (B) Panels show EGFP as green, CCR2 as red, F4/80 as blue, and the merged images of EGFP, CCR2, F4/80, and DIC images. White triangles, the yellow triangle, and the yellow asterisk indicate EGFP^+^F4/80^+^CCR2^+^ cells, an EGFP^+^F4/80^+^CCR2^–^ cell, and an EGFP^+^F4/80^−^CCR2^+^ cell, respectively. Scale bars, 30 µm. (C). Numbers of EGFP^+^F4/80^+^CCR2^+^ cells and EGFP^+^F4/80^+^CCR2^–^ cells in the pancreas of both groups. Data are the means ± SD of three mice per group. *P<0.05 versus mice fed a normal chow. HPF, high power field.

The injured pancreas contains several types of hematopoietic cells including neutrophils, lymphocytes and monocytes/macrophages. We analyzed the effect of irbesartan on leukocyte infiltration into the injured pancreas. EGFP^+^ BM-TNC-transplanted mice were fed a normal chow diet or an irbesartan-containing diet for 6 weeks. One week after the initiation of irbesartan treatment, CCl_4_ was intraperitoneally administered twice a week for 5 weeks (Figure 1D). Immunohistochemistry was used to identify Ly6G^+^ neutrophils, B220^+^ B cells, CD3ε^+^ T cells, and F4/80^+^ monocytic cells. Only EGFP^+^F4/80^+^ cells were reduced in the pancreas of irbesartan-treated mice, whereas no significant differences in the numbers of Ly6G^+^ cells, B220^+^ cells, and CD3ε^+^ cells were observed between control and irbesartan-treated mice (Figure 5A). We co-stained frozen pancreas sections with anti-F4/80 and anti-CCR2 antibodies, and identified two populations of monocytic cells, F4/80^+^CCR2^+^ cells and F4/80^+^CCR2^–^ cells. Two thirds of EGFP^+^F4/80^+^ cells were positive for CCR2 in the pancreas of control mice (Figure 5B, C). EGFP^+^F4/80^+^CCR2^+^ cells, but not EGFP^+^F4/80^+^CCR2^–^ cells, were significantly reduced by irbesartan treatment (Figure 5C). These results show that irbesartan inhibits the infiltration of CCR2^+^ monocytes into the injured pancreas.

**Figure 6:**
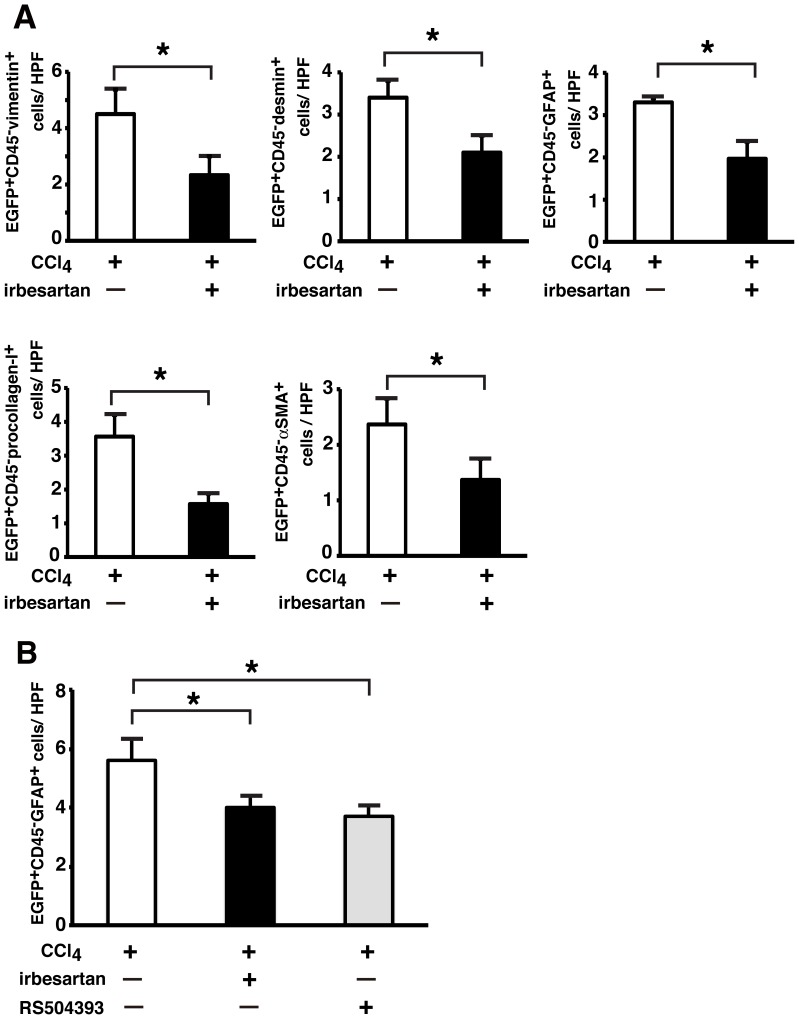
Effect of irbesartan on the occurrence of hematopoietic cell-derived PaSCs in pancreas of CCl_4_-treated mice. EGFP^+^ BM-TNC-transplanted mice were fed a normal chow diet or an irbesartan-containing diet for 6 weeks and treated with CCl_4_ for 5 weeks. (A) Numbers of EGFP^+^CD45^–^vimentin^+^, EGFP^+^CD45^–^desmin^+^, EGFP^+^CD45^–^GFAP^+^, EGFP^+^CD45^–^procollagen-I^+^, and EGFP^+^CD45^–^α-SMA^+^cells in the pancreas of both groups. Data are the means ± SD of three mice per group. *P<0.05 versus mice fed normal chow. (B) Comparison of irbesartan and RS504393 in the occurrence of EGFP^+^CD45^–^GFAP^+^ cells in the pancreas of CCl_4_-treated mice. Data are the means ± SD of three mice per group. *P<0.05 versus mice fed normal chow. HPF, high power field.

Next, we examined the effects of irbesartan on the occurrence of EGFP^+^ PaSCs in the pancreas of the EGFP^+^ BM-TNC-transplanted mice with CCl_4_-induced injury. As shown in Figure 6A, *in vivo* irbesartan treatment diminished the number of EGFP^+^CD45^–^vimentin^+^ cells by 52.9%, EGFP^+^CD45^–^desmin^+^ cells by 61.8%, EGFP^+^CD45^–^GFAP^+^ cells by 59.7%, EGFP^+^CD45^–^procollagen-I^+^ cells by 44.0%, and EGFP^+^CD45^–^α-SMA^+^ cells by 57.8%. Similar to the results obtained from the CCl_4_-injured chimeric mice treated with irbesartan, RS504393 also decreased the number of EGFP^+^ PaSCs in the pancreas of the mice (Figure 6B). The reduction of EGFP^+^CCR2^+^ monocytes in the CCl_4_-injured pancreas appeared to be associated with a parallel decrease in the number of EGFP^+^ PaSCs.

## Discussion

The origin of HpSCs and PaSCs has been a matter of much debate in recent years. Prior studies suggested that both types of stellate cells are derived from BM cells [39]–[44]. We previously reported that hematopoietic stem cell-derived cells could give rise to HpSCs [5]. The objective of the present study was to analyze whether hematopoietic lineage cells also differentiate into PaSCs using single EGFP^+^ hematopoietic stem cell-transplanted chimeric mice treated with CCl_4_. One third of EGFP^+^ cells in the pancreases were negative for CD45, and approximately 70% of EGFP^+^CD45^–^ cells were positive for stellate cell-associated antigens. When the proportions of EGFP^+^ PaSCs in the pancreases of the chimeric mice were compared with those of EGFP^+^ BM-TNC-transplanted mice, no statistically significant differences were observed. This indicated that a subset of PaSCs might be of hematopoietic origin. Furthermore, as EGFP^+^ PaSCs were detected in mice that received PB-TNCs or Ly6C^high^c-kit^–^ monocytes, but not in those that received a PB-Ly6C^+^ cell-depleted-population, PaSCs might be derived from terminally differentiated monocytes. As monocytes are extrahepatic HpSC precursors [4], we speculate that monocytes are common precursors of both HpSCs and PaSCs in CCl_4_-injured mice.

Recent studies reported that Ang II participates in MNC recruitment via the synthesis of MCP-1 by resident cells [45]–[48]. MCP-1 is considered an important chemotactic factor that regulates the migration of monocytes into the injured pancreas [14], [49], [50]. In our study, higher expression of MCP-1 and angiotensinogen was observed in the pancreases of CCl_4_-treated mice, compared with control mice. Isolated monocytes expressed both CCR2 and AT1R in our model [21], [51], [52]. Accordingly, we examined the mechanism of monocyte infiltration into the pancreas using the AT1R antagonist irbesartan that also acts as a CCR2 antagonist. Our *in vitro* results demonstrated that MCP-1 recruited freshly isolated Ly6C^+^ monocytes dose-dependently, and that irbesartan inhibited monocyte migration toward MCP-1 in a dose-dependent manner. Moreover, the numbers of EGFP^+^F4/80^+^CCR2^+^ monocytic cells and EGFP^+^ PaSCs in the pancreas decreased after treatment with irbesartan. Because irbesartan is known to act as not only an antagonist of AT1R and CCR2 but also a partial agonist of peroxisome proliferator-activated receptor gamma (PPAR-γ) [28], [53], the migration of monocytes into the injured pancreas may be prevented owing to its dual properties as a CCR2 antagonist and a PPAR-γ agonist. Furthermore, as Ang II-induced MCP-1 production in the pancreas is reduced by AT1R antagonists [54], irbesartan may also inhibit monocyte infiltration through the decrease of MCP-1. To clarify more precisely the contribution of CCR2 to monocyte migration, we used the selective CCR2 antagonist RS504393. Administration of RS504393 also inhibited *in vitro* Ly6C^+^ monocyte migration toward MCP-1 and the *in vivo* occurrence of EGFP^+^ PaSCs in the pancreas of EGFP^+^ BM-TNC-transplanted mice. Taken together, it is likely that CCR2^+^ monocytes migrate into the pancreas possibly via the MCP-1/CCR2 pathway and then differentiate into PaSCs. However, the present study did not clarify the mechanism of how engrafted monocytes give rise to PaSCs in the injured pancreas. Further studies are required to determine the generation process of PaSCs from monocytes.

## Supporting Information

Materials and Methods S1 **Supplementary materials and methods.**
(DOC)
