# Supplementary material for: Monocytes Infiltrate the Pancreas via the MCP-1/CCR2 Pathway and Differentiate into Stellate Cells
Source: PLoS One. 2014 Jan 8;9(1):e84889. doi: 10.1371/journal.pone.0084889 (PMC3885670; doi:10.1371/journal.pone.0084889)
Supplement: Materials and Methods S1 — Supplementary materials and methods. (DOC) [file pone.0084889.s001.doc]

# **Supplementary Materials and Methods S1**

## Antibodies and cytokines

Phycoerythrin (PE)-conjugated hamster anti-mouse CD3e, unconjugated rat anti-mouse CD4, unconjugated rat anti-mouse CD8a, biotinylated rat anti-mouse CD34, unconjugated rat ani-mouse CD45, PE-conjugated rat anti-mouse CD45.2, unconjugated or PE-conjugated rat anti-mouse CD45R/B220, unconjugated or allophycocyanin (APC)-conjugated rat anti-mouse c-kit, unconjugated or PE-conjugated rat anti-mouse Gr-1, unconjugated or PE-conjugated rat anti-mouse Ly6G, unconjugated or PE-conjugated rat anti-mouse Mac-1, unconjugated or PE-conjugated rat anti-mouse Sca-1, unconjugated rat anti-mouse TER-119, unconjugated or PE-conjugated rat anti-mouse Thy-1.2 and mouse lineage panel (biotinylated hamster anti-mouse CD3e, biotinylated rat anti-mouse CD45R/B220, biotinylated rat anti-mouse Gr-1, biotinylated rat anti-mouse Mac-1, and biotinylated rat anti-mouse TER-119) antibodies, streptavidin conjugated APC, streptavidin-conjugated APC-cyanine (Cy) 7, and APC-conjugated isotype control were purchased from BD Biosciences (San Diego, CA, USA). Polyclonal rabbit anti-angiotensin II receptor, type 1 (AT1R), polyclonal goat anti-desmin, polyclonal goat anti-procollagen 1A1, and polyclonal rabbit anti-vimentin antibodies were from Santa Cruz Biotechnology (Santa Cruz, CA, USA). Other antibodies were as follows: polyclonal goat anti-C-C chemokine receptor 2 (CCR2) antibody (Genetex, Los Angeles, CA, USA), unconjugated rat anti-mouse CCR3 antibody (R&D systems, Minneapolis, MN, USA), APC-conjugated rat anti-mouse CD45 antibody (eBioscience, San Diego, CA, USA), APC-conjugated rat anti-mouse F4/80 antibody (Caltag, Burlingame, CA, USA), polyclonal rabbit anti-cow glial fibrillary acidic protein antibody (Dako Cytomation, Carpinteria, CA, USA), biotinylated or PE-conjugated rat anti-mouse Ly6C antibody and anti-PE microbeads (Miltenyi Biotec, Auburn, CA, USA), Cy3-conjugated monoclonal mouse anti-α-smooth muscle actin  antibody (Sigma-Aldrich, St. Louis, MO, USA), Cy3-conjugated mouse IgG (Jackson Immunoresearch Laboratories, West Grove, PA, USA), and Alexa Fluor 568- and Alexa Fluor 647-conjugated secondary antibodies against primary antibodies of rat, rabbit or goat origin (Molecular Probes Invitrogen, Eugene, OR, USA). Recombinant mouse (rm) steel factor, rm monocyte chemoattractant protein-1 (MCP-1), rm macrophage inflammatory protein-1α, and recombinant human interleukin-11 were purchased from R&D Systems. Human angiotensin II was purchased from Sigma-Aldrich.

## RT-PCR

Total RNA was extracted from whole mouse pancreas and Ly6C^+^ monocytes, which were isolated from BM-MNCs, using TRIzol reagent (Invitrogen) according to the manufacturer’s instructions. For RT-PCR, 4 μg total RNA was reverse-transcribed to cDNA using random hexamer primers and Ready-to-Go You-Prime First-Strand Beads (GE Healthcare, Buckinghamshire, UK). cDNA was amplified by PCR using Taq DNA polymerase (Takara Bio, Shiga, Japan) and the following primers: mouse MCP-1 (sense primer, 5’-ATGCAGGTCCCTGTCATG-3’ and antisense primer, 5’-GCTTGAGGTGGTTGTGGA-3’), mouse angiotensinogen (sense primer, 5’-TGAATGAGGCAGGAAGTG-3’ and antisense primer, 5’-AGGCTCTGAACAAATGAGTG-3’), mouse CCR2 (sense primer, 5’-GGTCATGATCCCTATGTGG-3’ and antisense primer,　5’-CTGGGCACCTGATTTAAAGG-3’), mouse AT1Ra (sense primer, 5’-TCACCTGCATCATCATCTGG-3’ and antisense primer, 5’-AGCTGGTAAGAATGATTAGG-3’), and mouse AT1Rb (sense primer, 5’-TGGCTTGGCTAGTTTGCCG-3’ and antisense primer, 5’-ACCCAGTCCAATGGGGAGT-3’). Primers specific for mouse glyceraldehyde-3-phosphate dehydrogenase (GAPDH) (sense primer, 5’-ACCACAGTCCATGCCATCAC-3’ and antisense primer, 5’-TCCACCACCCTGTTGCTGTA-3’) were used as an internal PCR control. PCR conditions were as follows: initial denaturation for 3 minutes at 94°C, followed by 40 cycles of denaturation for 30 seconds at 94°C, annealing for 30 seconds at 65°C (MCP-1), 54°C (angiotensinogen), 50°C (CCR2) or 58°C (AT1Ra, AT1Rb and GAPDH), and extension for 30 seconds at 72°C. PCR products were analyzed by 2% agarose gel electrophoresis with ethidium bromide staining. The sizes of the amplified DNA bands for GAPDH, MCP-1, angiotensinogen, CCR2, AT1Ra, and AT1Rb were 500, 429, 342, 328, 224, and 140 base pairs, respectively. DNA bands were semiquantitatively evaluated with image analysis software (NIH Image 1.61, Bethesda, MD, USA), and the intensities of MCP-1 and angiotensinogen were normalized to that of GAPDH.
